# Encapsulation of α-Pinene in Delivery Systems Based on Liposomes and Cyclodextrins

**DOI:** 10.3390/molecules26226840

**Published:** 2021-11-12

**Authors:** Zahraa Hammoud, Maya Kayouka, Adriana Trifan, Elwira Sieniawska, Jouda Mediouni Ben Jemâa, Abdelhamid Elaissari, Hélène Greige-Gerges

**Affiliations:** 1 Bioactive Molecules Research Laboratory, Doctoral School of Sciences and Technologies, Faculty of Sciences, Lebanese University, Jdeidet El-Metn 90656, Lebanon; zahraahammoud93@gmail.com (Z.H.); maya.kayouka@gmail.com (M.K.); 2 UMR-5280, CNRS-University Lyon-1, 5 rue de la Doua, 69100 Villeurbanne, France; abdelhamid.elaissari@univ-lyon1.fr; 3 Department of Pharmacognosy, Faculty of Pharmacy, Grigore T. Popa University of Medicine and Pharmacy of Iasi, 16, 700115 Iasi, Romania; adriana.trifan@umfiasi.ro; 4 Department of Natural Products Chemistry, Medical University of Lublin, 20-093 Lublin, Poland; 5 Laboratory of Biotechnology Applied to Agriculture, National Agricultural Research Institute of Tunisia (INRAT), University of Carthage, El Menzah 1004, Tunisia; j_mediouni@hotmail.fr

**Keywords:** α-pinene, drug-in-cyclodextrin-in-liposomes, encapsulation, hydroxypropyl-β-cyclodextrin, liposomes

## Abstract

The essential oil component α-pinene has multiple biological activities. However, its application is limited owing to its volatility, low aqueous solubility, and chemical instability. For the aim of improving its physicochemical properties, α-pinene was encapsulated in conventional liposomes (CLs) and drug-in-cyclodextrin-in-liposomes (DCLs). Hydroxypropyl-β-cyclodextrin/α-pinene (HP-β-CD/α-pinene) inclusion complexes were prepared in aqueous solution, and the optimal solubilization of α-pinene occurred at HP-β-CD:α-pinene molar ratio of 7.5:1. The ethanol-injection method was applied to produce different formulations using saturated (Phospholipon 90H) or unsaturated (Lipoid S100) phospholipids in combination with cholesterol. The size, the phospholipid and cholesterol incorporation rates, the encapsulation efficiency (EE), and the loading rate (LR) of α-pinene were determined, and the storage stability of liposomes was assessed. The results showed that α-pinene was efficiently entrapped in CLs and DCLs with high EE values. Moreover, Lipoid S100 CLs displayed the highest LR (22.9 ± 2.2%) of α-pinene compared to the other formulations. Both carrier systems HP-β-CD/α-pinene inclusion complex and Lipoid S100 CLs presented a gradual release of α-pinene. Furthermore, the DPPH radical scavenging activity of α-pinene was maintained upon encapsulation in Lipoid S100 CLs. Finally, it was found that all formulations were stable after three months of storage at 4 °C.

## 1. Introduction

Essential oils and their components have been known to possess antioxidant and antimicrobial activities. In recent years, the uses of essential oils as natural additives for the shelf-life extension of food products have received increasing attention, due to the risk of using synthetic preservatives [1]. Alpha-pinene, also called 2,6,6-trimethylbicyclo [3.1.1] hept-2-ene, is a natural bicyclic monoterpene [2]. It is a component of the essential oils of rosemary (*genus Rosmarinus*, *species Rosmarinus officinalis* L.) and several coniferous trees from the Pinaceae (*genus Pinus*) and Lamiaceae family (e.g., *lavender*, *genus Lavandula*) [3,4]. The market volume of α-pinene is 22 million kg per year and the price is approximately 2.5 USD per 1 Kg [5]. It has been demonstrated that α-pinene possesses insecticidal [6], antioxidant [7], antimicrobial [8,9], anti-inflammatory [10], and anti-tumor [11] effects. In addition, α-pinene is generally recognized as safe (GRAS) by the Food and Drug Administration and other regulatory agencies [12]. Thus, it can be used in the preparation of a wide range of value-added products in the agricultural and food sectors.

**Figure 1:**
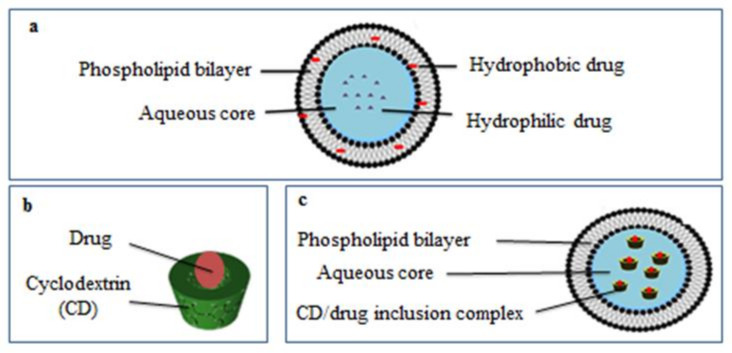
The structure of a liposome (**a**), cyclodextrin/drug inclusion complex (**b**) and drug-in-cyclodextrin-in-liposomes (**c**).

The low aqueous solubility (about 2.5 mg/L at 25 °C), high photosensitivity, and high volatility restrict the application of α-pinene [13,14,15]. The development of encapsulation systems loading α-pinene can be utilized to improve the physicochemical properties of the compound and to allow its controlled release from the fabricated matrices [16]. Attempts were made to improve the solubility and the stability of α-pinene through encapsulation in solid lipid nanoparticles [2], chitosan:gum Arabic microcapsules [17], or cyclodextrins (CDs) [18,19,20]. CDs, non-toxic cyclic oligosaccharides having a basket-shaped structure with a hydrophilic outer surface and a hydrophobic cavity, increased the solubility and the stability of hydrophobic drugs, including α-pinene, by forming CD/drug inclusion complexes. Figure 1 represents the structure of a CD/drug inclusion complex. Compared to α-CD and γ-CD, β-CD and its derivatives (hydroxypropyl-ß-cyclodextrin (HP-β-CD), randomly methylated-β-cyclodextrin, and a low methylated-β-cyclodextrin) were shown to be very effective for α-pinene encapsulation. Moreover, the complexation efficiency of native β-CD is close to that of β-CD derivatives [18].

In parallel, liposomes are specialized delivery vehicles that serve multiple roles in improving the effectiveness of bioactive molecules. They are non-toxic microscopic vesicles with concentric phospholipid bilayers surrounding an inner aqueous core. This structure makes liposomes suitable for entrapping hydrophobic, hydrophilic, and amphiphilic substances, as shown in Figure 1 [21].

The drug-in-cyclodextrin-in-liposomes (DCLs) system is based on the entrapment of lipophilic drugs in the aqueous compartment of liposomes in the form of CD/drug inclusion complexes (Figure 1). This system combines the relative advantages of both carriers [22]. DCLs improved the encapsulation of essential oil components such as eugenol [23], trans-anethole [24], estragole [25,26], and thymol [26], delayed drug release [25,26,27], and prolonged the biological effect of the encapsulated compounds [28,29] in comparison to conventional liposomes (CLs).

CDs are capable of removing phospholipids and cholesterol (Chol) from the liposomal membrane, thereby perturbing some of its properties such as its permeability and fluidity [30,31,32]. The impact of CD on lipid membranes is mediated by several factors including membrane structure and composition as well as the CD type and the CD concentration [33,34,35]. HP-β-CD, which was used in the present study to prepare the CD/α-pinene inclusion complex, does not influence the integrity of liposome membranes compared to other CD types [36,37].

In this study, we aimed to develop an appropriate formulation for the effective delivery of α-pinene that can be used to promote the biological effects of the molecule, particularly its insecticidal effect. For this purpose, α-pinene was encapsulated into CL and DCL systems composed of hydrogenated (Phospholipon 90H) or non-hydrogenated (Lipoid S100) phospholipids using the ethanol-injection method. All the formulations were characterized for their size using laser granulometry. Furthermore, the incorporation rate of phospholipids and cholesterol as well as the encapsulation efficiency and the loading rate of α-pinene were calculated. Moreover, the antioxidant activity of free and encapsulated α-pinene was evaluated using a 2,2-diphenyl-1-picrylhydrazyl (DPPH•) scavenging assay. Finally, the release of α-pinene from the inclusion complex and from Lipoid S100-CLs was analyzed using dialysis tubing, and the stability of the liposome formulations in aqueous suspensions was assessed after three months of storage at 4 °C.

## 2. Results and Discussion

### 2.1. Determination of α-Pinene Concentration in HP-β-CD/α-Pinene Inclusion Complex Solutions

**Figure 2:**
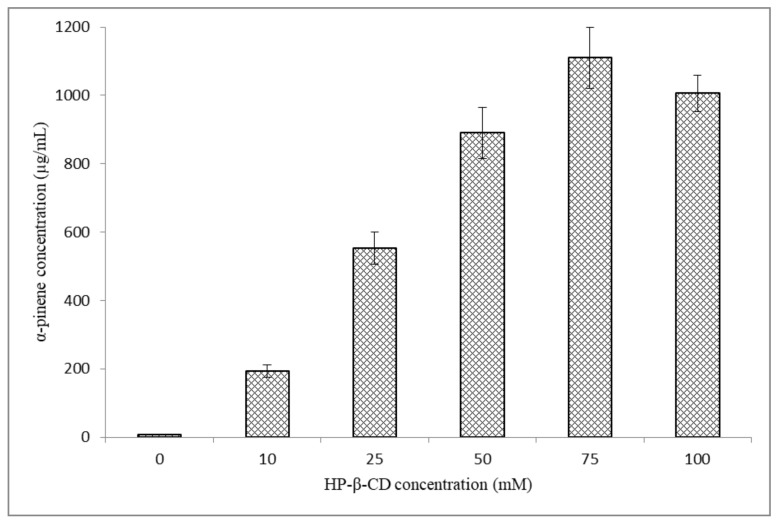
The variation of α-pinene concentration in the filtrate as a function of HP-β-CD concentration.

HP-β-CD/α-pinene inclusion complexes were prepared in aqueous solution, and the optimal HP-β-CD:α-pinene molar ratio was determined. Figure 2 shows the variation of α-pinene concentration in HP-β-CD/α-pinene complex solutions at various HP-β-CD concentrations (0–100 mM). In the absence of HP-β-CD, the concentration of α-pinene was 3.2 ± 0.1 µg/mL. This value is in accordance with the value of aqueous solubility found in the literature (2.5 µg/mL) [38]. The addition of HP-β-CD leads to an enhancement of α-pinene solubility up to 366 times at HP-β-CD concentration of 75 mM. CE values permitted the evaluation of the optimal HP-β-CD:α-pinene molar ratio. The CE of α-pinene into HP-β-CD was determined as explained in equation 1. The results are reported in Table 1. The CE of α-pinene was 14.3 ± 1.3% at HP-β-CD:α-pinene molar ratio of 1:1, and the CE increased to 80.6 ± 7.7% at HP-β-CD:α-pinene molar ratio of 7.5:1. Nevertheless, further increase in HP-β-CD concentration did not improve the CE of α-pinene. Consequently, the HP-β-CD/α-pinene inclusion complex prepared at HP-β-CD:α-pinene molar ratio of 7.5:1 was selected as the aqueous phase for the preparation of DCL batches.

### 2.2. Characterization of Liposome Formulations

#### 2.2.1. Determination of Liposome Particle Size

The particle size of the liposomal batches was measured using a laser granulometer and presented in Figure S1 (Supplementary materials). Table 2 represents the percentage distribution and the mean particle size of the populations in each formulation.

Blank Phospholipon 90H:Chol CLs presented two distinct populations: a nanometric sized population with a mean size of 150 ± 0.0 nm and a micrometric sized population with a mean size of 6.2 ± 0.9 µm; the percentage of the nanometric population was 87.7 ± 6.7%. The results are in good agreement with the results of Azzi et al. (2018) [39]. Moreover, it was found that the incorporation of α-pinene in Phospholipon 90H:Chol liposome did not affect the mean vesicle size and the size distribution of the blank batch.

For blank Lipoid S100:Chol CLs, two populations with nanometric (166 ± 33.3 nm) and micrometric (6.7 ± 0.0 µm) sizes were observed. The micrometric population was the major one since it represents 83.0 ± 1.4% of the vesicles. The results are in accordance with our previous study [40]. Compared to blank Lipoid S100:Chol CLs, α-pinene boosted the formation of larger micrometric vesicles. The accumulation of α-pinene in the Lipoid S100 liposome membrane may affect the interactions between the acyl chains of phospholipids and induce swelling of the membrane, causing the formation of giant particles [41]. Rodriguez et al. (2018) reported that the incorporation of α-pinene in the DPPC lipid membrane induced an increase in the membrane surface pressure and the area per lipid due to the interaction of α-pinene with the acyl chain of lipids forming lipid-α-pinene complex [42]. It is noteworthy that many other essential oil components induce an increase in liposome particle size such as eugenol [23], nerolidol [27], isoeugenol, and thymol [40].

The addition of HP-β-CD and HP-β-CD/α-pinene inclusion complex did not significantly influence the mean vesicle size of blank Phospholipon 90H:Chol formulation, which is in agreement with other studies [24,25,28,29].

HP-β-CD modified the size distribution of blank Lipoid S100:Chol liposomes. A larger population was obtained after the addition of HP-β-CD to lipoid S100 liposomes that is in accordance with our previous results obtained at HP-β-CD concentrations of 25 and 75 mM [26]. Moreover, it was previously demonstrated that the addition of HP-β-CD induced an increase in the fluidity of liposome membrane composed of unsaturated phospholipids and Chol (Lipoid S100:Chol liposomes) while did not influence that of membranes composed of saturated phospholipid and Chol (Phospholipon 80H:Chol and Phospholipon 90H:Chol liposomes) [43]. Alpha-pinene-DCLs had a smaller particle size compared to α-pinene-CLs and HP-β-CD-loaded liposomes. When α-pinene was entrapped in HP-β-CD core, the effects of both constituents (α-pinene and HP-β-CD) on liposome membrane were reduced. The same results were reported for the encapsulation of HP-β-CD/eugenol [23], HP-β-CD/isoeugenol, and HP-β-CD/thymol inclusion complexes [26] in liposomes when compared to free HP-β-CD and free guests.

#### 2.2.2. Determination of Phospholipid: Chol:α-Pinene Molar Ratio

Phospholipids and Chol IR values, α-pinene EE, and α-pinene LR into the various liposome formulations were determined. Table 2 summarizes the obtained results.

##### Phospholipid Incorporation Rate

The dosage of phospholipids proved a high IR for Phospholipon 90H (89.5 ± 6.3%) and Lipoid S100 (95.9 ± 1.5%) in blank liposomes. The addition of α-pinene did not greatly influence the loading of Phospholipon 90H in the liposome membrane while it reduced that of Lipoid S100. The difference could be attributed to the amount of α-pinene loaded in liposome membrane; the LR value of α-pinene was 0.2 ± 0.01% into Phospholipon 90H vesicles and 22.9 ± 2.2% into Lipoid S100 vesicles.

In the presence of HP-β-CD, the IR of Phospholipon 90H and Lipoid S100 in liposomes markedly decreased compared to the blank formulations, which is in agreement with our previous investigations [26]. This finding might be explained by the ability of CDs to interact with lipid bilayers and to mediate the desorption of lipid components from membranes [30].

In comparison to the blank batches, we noticed that the HP-β-CD/α-pinene inclusion complex did not greatly impact the incorporation of phospholipids (either Phospholipon 90H or Lipoid S100) in liposome membranes. This finding implies that α-pinene is completely loaded into the lipophilic core of HP-β-CD, and this is in accordance with the results demonstrating no effects of the inclusion complex on the particles size.

##### Cholesterol Incorporation Rate

Chol quantification assay showed that the IR values of Chol into blank Phospholipon 90H liposomes and blank Lipoid S100 liposomes were 75.9 ± 3.5% and 71.2 ± 5.4%, respectively. These values are approximately consistent with recently published data: 84.2 ± 3.8% into Phospholipon 90H liposomes [39] and 77.6 ± 1.18% into Lipoid S100 liposomes [26,40]. Alpha-pinene did not modify the Chol content of Phospholipon 90H and Lipoid S100 liposome membranes.

A remarkable decrease in the Chol IR was obtained upon the addition of HP-β-CD to Phospholipon 90H liposomes in comparison to empty vesicles (Table 2). This is determined for the first time in literature. Furthermore, the addition of HP-β-CD did not significantly influence the retention of Chol into Lipoid S100 liposomes in agreement with our previously published data [26]. On the other hand, the loading of HP-β-CD/α-pinene inclusion complex into liposome cavity lowered Chol entrapment in Phospholipon 90H liposomes while it did not drastically impact the Chol content of Lipoid S100 liposomes in comparison to the blank liposomes. Hence, HP-β-CD may extract Chol molecules from the membrane of Phospholipon 90H:Chol vesicles forming soluble inclusion complexes out of the membrane, while no effect was exerted on Lipoid S100 vesicles. Yancey et al., (1996) proposed that the CD-mediated Chol extraction occurs by Chol desorption from the surface directly into CD hydrophobic core [44]. In addition, Steck et al. (2002) suggested that Chol efflux induced by CDs takes place through an activation-collision mechanism where the reversible partial projection of Chol molecules out of the erythrocyte lipid bilayer precedes their collisional capture by CD [45]. On the other hand, the position and orientation of Chol in saturated and unsaturated lipid bilayers have been widely studied in the literature [46,47,48]. A common conclusion is that in saturated bilayers, Chol is found in traditional “upright” positions with the hydroxyl group oriented towards lipid head-groups, while in unsaturated bilayers, Chol is relatively often found in a “flipped” configuration with the hydroxyl group oriented towards the membrane middle plane. Furthermore, Chol molecules in unsaturated bilayers are often found to form head-to-tail contacts which may lead to specific clustering behavior. For that, CD-mediated Chol extraction is easier from saturated Phospholipon 90H:Chol membranes compared to unsaturated Lipoid S100:Chol membranes.

##### α-Pinene Encapsulation Efficiency and Loading Rate

Due to the fact that α-pinene is a highly lipophilic compound, it should be entrapped in the lipid membrane of liposomes. Therefore, the strength of interaction of α-pinene with the lipid membrane of liposomes influences its incorporation in vesicles. Furthermore, if we suppose that the hydrophobic compartments of DCLs (CD core and liposome membrane) retain the hydrophobic EO components, the parameters that modulate α-pinene entrapment into DCL vesicles are those related to CD-α-pinene interaction and lipid membrane-α-pinene interaction. The EE and LR of α-pinene into CLs and DCLs were determined, and the results are summarized in Table 2.

Alpha-pinene was highly encapsulated into all CL and DCL formulations. EE values of 100% were obtained for all CL formulations and for Lipoid S100-DCLs; the EE of α-pinene into Phospholipon 90H-DCLs was 72.9 ± 6.8%. The high affinity of α-pinene for both components (CDs and lipid bilayers) could explain the findings; the interaction of α-pinene with CD and lipid bilayer is strong enough to inhibit the release of α-pinene to the outer extracellular medium of CL and DCL systems. Indeed, it was reported that α-pinene possesses a high affinity for DPPC lipid membrane due to its high hydrophobicity and chemical structure that does not contain polar groups. Thus, α-pinene cannot interact directly with the DPPC polar head but it is able to interact with the hydrophobic tails of the lipids. Namely, this small hydrophobic compound adopts a position in the acyl region of the membrane rather than at the lipid-water interface [42]. Besides, it was investigated that the loading of hydrophobic drugs into liposomes increases as the hydrophobicity of the drug increases [40,49]. On the other hand, the strength of CD-guest interaction depends mainly on the polarity and geometric accommodation between CD cavity and guest [50]. Here, the low molecular weight (136.23 g/mol) and the high hydrophobicity of α-pinene could explain the strong interaction of α-pinene with CD. Moreover, it should be noted that the stability of the inclusion complexes modulates the retention of EO components into DCLs. The formation constant (Kf) values reported in literature for HP-β-CD/α-pinene inclusion complex were 1637 M-1 [18] and 2000 M-1 [19].

Among both types of phospholipids, the highest LR value was obtained with Lipoid S100, where the LR value of α-pinene into Lipoid S100 liposomes (22.9 ± 2.2%) was approximately 100 times higher than that of Phospholipon 90H liposomes (0.2 ± 0.02%). Lipid bilayers composed of Lipoid S100 (unsaturated phospholipid) are less densely packed and more flexible compared to those composed of Phospholipon 90H (saturated phospholipid), leading to greater entrapment of α-pinene into liposome membrane [51]. Furthermore, Lipoid S100 liposomes were fabricated at room temperature while the preparation of Phospholipon 90H liposomes requires heating at 55 °C, thus promoting the loss of volatile α-pinene.

When compared to α-pinene loaded-Phospholipon 90H-CLs, the DCL carrier system did not ameliorate the LR of α-pinene. Additionally, concerning Lipoid S100 formulations, the LR value of α-pinene into DCLs was lower than that of CLs. The LR values into CLs and DCLs were 22.9 ± 2.2% and 0.6 ± 0.02%, respectively (Table 2). Although this result does not corroborate the previous studies where DCL improved the loading of essential oil components compared to CLs [23,24,52,53], it lies with the encapsulation of risperidone into similar formulations (same lipid composition and CD type) where CL proved better encapsulation than DCL [54].

The final liposome composition (phospholipid:Chol:α-pinene molar ratio) was 124:98:0.36, 118:82:0.31, 61:93:42 and 111:92:1 for phospholipon 90H-CLs, phospholipon 90H-DCLs, Lipoid S100-CLs, and Lipoid S100-DCLs, respectively. Based on the characteristics of CLs and DCLs, Lipoid S100:Chol-CL was the best encapsulating system for α-pinene compared to the other formulations. This explains the greatest effect of α-pinene on the Lipoid S100-CL membrane compared to the other formulations.

#### 2.2.3. In Vitro Release Study

**Figure 3:**
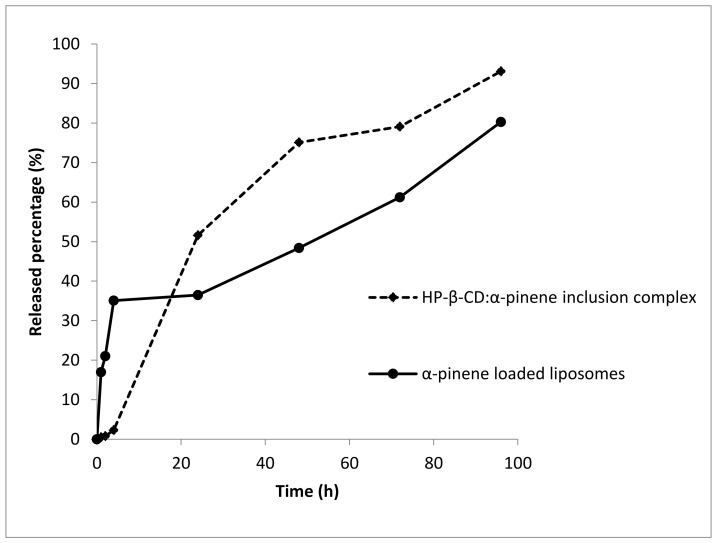
The in vitro release profile of α-pinene from HP-β-CD:α-pinene inclusion complex and from Lipoid S-100 liposomes.

The release study was performed using dialysis tubing. It should be noted that the release studies of α-pinene from Lipoid S100-DCLs, Phospholipon 90H-CLs, and Phospholipon 90H-DCLs were not considered since the amount of α-pinene in these formulations was very low. The in vitro release profiles of α-pinene from HP-β-CD/α-pinene inclusion complex and from Lipoid S100-CLs are shown in Figure 3. The results are expressed as the percentage of α-pinene released from the formulation as a function of time. We could notice from Figure 3 that α-pinene was gradually released from the HP-β-CD/α-pinene inclusion complex and Lipoid S100 liposomes. Even though the release of α-pinene from the liposome formulation was faster than the inclusion complex during the first hours of the study (first 6 h), liposomes showed a higher ability to retain α-pinene for the rest of the kinetics. To sum up, CDs and liposomes could retain and allow a controlled release for α-pinene.

#### 2.2.4. Determination of DPPH Radical Scavenging Activity

**Figure 4:**
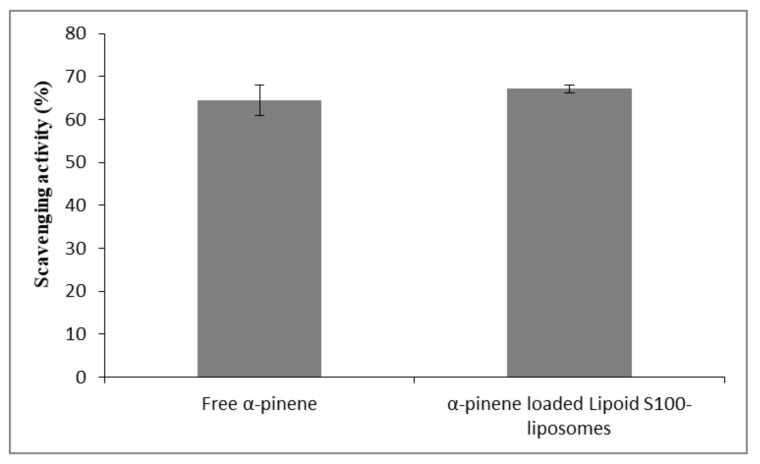
DPPH• scavenging activities of free α-pinene and α-pinene-loaded Lipoid S-100 liposomes measured at α-pinene concentration of 200 µg/mL.

To evaluate the in vitro antioxidant capacity of α-pinene and α-pinene-loaded Lipoid S-100 liposomes, we used DPPH• scavenging assay. The free radical scavenging activity was expressed as the percentage inhibition of DPPH, and the results are represented in Figure 4. A good radical scavenging activity against DPPH was observed for α-pinene (64.4%). This finding is in line with the literature data [7,8,55,56]. As can be seen in Figure 4, the DPPH• scavenging activity of free α-pinene was kept unchanged upon being loaded in Lipoid S100 liposomes. This indicates that Lipoid S100 liposome formulations are able to preserve the antioxidant activity of α-pinene. In the conditions of the experiments, equal volumes of water and ethanol are present allowing the complete solubilization of DPPH and alpha-pinene. Even though alpha-pinene is entrapped in liposomes, its ability to scavenge radicals is not prohibited. This finding was documented for other antioxidant products in their free and encapsulated forms in liposomes [57,58]. As mentioned before, the amount of α-pinene in Lipoid S100-DCLs, Phospholipon 90H-CLs, and Phospholipon 90H-DCLs was very low; thus the antioxidant capacity of these formulations was not evaluated. On the other hand, the DPPH• scavenging assay of the CD/α-pinene inclusion complex was not studied due to the possibility of inclusion of DPPH• inside the CD cavity [59]. Moreover, the high ethanol to water ratio (2:1, *v/v*) may lead to the dissociation of the HP-β-CD/α-pinene inclusion complex [60].

#### 2.2.5. Storage Stability

The storage stability of the various nanoparticles was evaluated after storage for three months at 4 °C in terms of size and percentage of remaining α-pinene in the whole suspension. All of the prepared batches, except the blank and α-pinene-Phospholipon 90H CLs, presented an increase in the liposome particle size and in the percentage of the largest population after three months (Table 2). The aggregation of particles during storage cannot be excluded. This could be attributed to the low colloidal stability of liposomes. The presence of surface charge prevents aggregation due to electrostatic repulsion, and the more neutral net charge resulted in the formation of aggregates [61]. The magnitude of zeta potential, which gives a prediction of the colloidal stability, was previously determined for blank Phospholipon 90H and Lipoid S100 liposomes. The authors demonstrated that both types of formulations possessed a low zeta potential value, and thereby are susceptible to aggregate [51].

**Figure 5:**
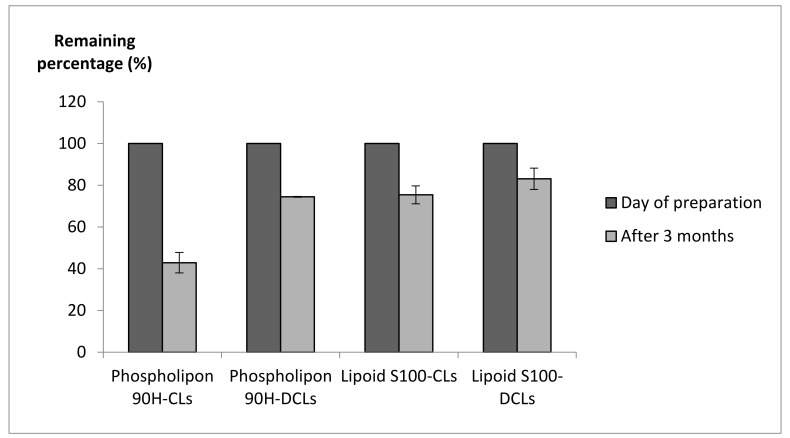
The percentage of remaining α-pinene determined on the day of preparation and after 3 months of storage at 4 °C.

Additionally, the total α-pinene concentration in the liposome suspensions was determined after three months by HPLC, and the values were compared to those obtained on the day of preparation. The percentage of remaining α-pinene was 42.9 ± 4.9%, 74.4 ± 0.2%, 75.4 ± 4.3%, and 83.1 ± 5.1% in Phospholipon 90H-CLs, Phospholipon 90H-DCLs, Lipoid S100-CLs, and Lipoid S100-DCLs, respectively (Figure 5). Hence, α-pinene was still satisfactorily incorporated into liposomes after three months, and the DCL carrier system was more effective in retaining α-pinene during the storage in comparison to CLs. Therefore, these formulations could be tested for their effectiveness in food products. For long-term storage, freeze-drying of liposomes in the presence of cyclodextrins can be performed as described previously [62].

## 3. Materials and Methods

### 3.1. Materials

Alpha-pinene was purchased from Acros Organics, Germany; cholesterol (Chol) (94%) from Acros organics, GEEL, Belgium; and p-cymene from Fluka, Buch, Switzerland. HP-ß-CD (DS = 5.6) was supplied by Roquette (Lestrem, France). Ammonium molybdate, potassium phosphate monobasic (KH2PO4), and 2,2-diphenyl-1-picrylhydrazyl (DPPH•) were purchased from Sigma-Aldrich, Darmstadt, Germany; methanol HPLC grade from Sigma-Aldrich, France; and triton X-100 from Sigma–Aldrich, St. Louis, MI, USA. Sulfuric acid and absolute ethanol were obtained from VWR Pro-labo chemicals, Fontenay-sous-Bois, France; 4-amino-3-hydroxy-1-naphthalenesulfonic acid from Fluka, Mumbai, India; and hydrogen peroxide from Fisher Scientific, Loughborough, UK. Chol CHOD-POD kit was purchased from Spin react Company, Girona, Spain. Phospholipon 90H (90% soybean PC, 4% lysoPC, 2% triglycerides, 2% water, 0.5% ethanol, iodine value 1) and Lipoid S100 (94% soybean phosphatidylcholine, 3% lysophosphatidylcholine, 0.5% N-acyl phosphatidylethanolamine, 0.1% phosphatidylethanolamine, 0.1% phosphatidylinositol, 0.2% ethanol, 2% water) were supplied by Lipoid GmbH, Ludwigshafen, Germany. Dialysis membranes (MWCO 0.5–1 kDa and 8–10 kDa) were obtained from Spectrum Laboratories (Rancho Dominguez, CA, USA).

### 3.2. HPLC Analysis of α-Pinene

Stock solutions of α-pinene (1 mg/mL) and of the internal standard p-cymene (1 mg/mL) were prepared in methanol. The stock solution of α-pinene was diluted to obtain final concentrations in the range of 1–100 μg/mL. The diluted solution of p-cymene (10 μg/mL) was also prepared in methanol. For HPLC analysis, 100 μL of each α-pinene solution was added to a solution of p-cymene (100 μL) and methanol (200 μL). The analysis was performed using an Agilent HPLC column (C18, 15 cm × 4.6 mm, 5 μm). The mobile phase was a mixture of methanol and water (85:15). The flow rate was fixed at 1 mL/min, and the injection volume was 20 μL. The detection was set at 204 nm.

The HPLC method was validated in terms of linearity, repeatability, and limit of detection. The retention times of p-cymene and α-pinene were 5.7 and 9.5 min, respectively. The calibration curve was constructed by plotting AUC_(α-pinene)/AUC_(p-cymene) against the concentration of α-pinene in μg/mL. The linear relationships were evaluated by regression analysis with the least-squares method, and the correlation coefficient ranged between 0.994 and 0.999.

### 3.3. Determination of the Optimal HP-β-CD Concentration for α-Pinene Solubilization

Excess amount of α-pinene (6.81 mg) was added to 5 mL of HP-β-CD solutions (0, 10, 25, 50, 75, 100 mM) yielding different HP-β-CD:α-pinene molar ratios (1:1, 2.5:1, 5:1, 7.5:1 and 10:1). The mixtures were shaken at a stirring rate of 125 rpm for 24 h at 26 °C. Then, to remove the un-dissolved α-pinene, the solutions were filtered through a 0.45 μm membrane filter. The amount of solubilized α-pinene was determined in the filtrate by HPLC analysis. The complexation efficiency (CE) was calculated using the following equation:

CE (%) = m_exp/m_the × 100(1)

in which mexp is the mass of α-pinene experimentally determined in the filtrate by HPLC. mthe is the mass of α-pinene initially used to prepare the inclusion complexes.

### 3.4. Preparation of HP-β-CD/α-Pinene Inclusion Complex for DCL Preparations

HP-β-CD/α-pinene inclusion complex of 7.5:1 (HP-β-CD:α-pinene) molar ratio was prepared by adding α-pinene to the HP-β-CD aqueous solution of 75 mM concentration. The mixture was stirred at a stirring rate of 125 rpm for 24 h at 26 °C. It was then filtered using a 0.45 μm cellulose filter. The filtrate was used for DCL preparation. Furthermore, the HP-β-CD aqueous solution of 75 mM concentration was used for the fabrication of blank DCLs.

### 3.5. Preparation of Liposomes

The liposomes were prepared by the ethanol-injection method. The appropriate amounts of phospholipids (Phospholipon 90H or Lipoid S100) (10 mg/mL) and Chol (5 mg/mL) were dissolved in absolute ethanol (10 mL) by stirring. The resulting organic phase was then injected into an aqueous solution (20 mL) using a syringe pump (Fortuna optima, Mannheim, Germany) at an injection flow rate of 1 mL/min, under magnetic stirring (400 rpm) and at a temperature above the transition temperature of phospholipids: 55 °C for Phospholipon 90H and room temperature for Lipoid S100. The liposomal suspensions were then maintained for 15 min under stirring (400 rpm) at room temperature. Finally, ethanol was evaporated under reduced pressure at 40 °C (Heidolph instruments, Schwabach, Germany). According to a previous study, the remaining ethanol percentage was determined in liposomes and DCL formulations and the results showed that the percentage of ethanol in suspensions did not exceed 3% [39].

Four liposomal batches were produced: (1) blank conventional liposomes (blank CLs), (2) blank DCLs, in which HP-β-CD (75 mM) was dissolved in the aqueous phase; (3) α-pinene-loaded liposomes (α-pinene-CLs), where α-pinene was dissolved in the organic phase at a concentration of 2.5 mg/mL; (4) HP-β-CD/α-pinene inclusion complex-loaded liposomes (α-pinene-DCLs), where a solution containing the HP-β-CD/α-pinene inclusion complex of 7.5:1 (HP-β-CD:α-pinene) molar ratio was used as the aqueous phase. Each batch was prepared in triplicate.

### 3.6. Characterization of Liposomes

#### 3.6.1. Particle Size Analysis

Laser granulometry (Partica Laser scattering, LA-950V2 particle size distribution analyzer, HORIBA, Japan) was used to determine the diameter and size distribution of particles. This instrument is designed for measuring particle sizes ranging from 0.01 μm to 3000 μm.

#### 3.6.2. Determination of Phospholipid:Chol:α-Pinene Molar Ratio in the Formulations

The concentrations of phospholipids, Chol, and α-pinene embedded in the lipid vesicles were calculated by subtracting the free concentrations of compounds from their total concentrations determined in the liposome suspensions. Liposome suspensions were centrifuged at 21,382 g and 4 °C for 1 h using a Vivaspin 500 centrifugal concentrator (Sartorius Stedim Biotech, Germany, MW cut off = 10,000 Da). The filtrate contains the unloaded (free) compounds. For each formulation, the final liposomal composition (phospholipid:Chol:α-pinene molar ratio) was determined.

##### Determination of Phospholipid Incorporation Rate

The total and the unloaded phospholipid concentrations in the various liposome formulations were quantified according to Bartlett’s method as described in our previous studies [26,40]. Standard aqueous solutions of phosphorus were prepared. The organic phosphate in the samples (0.5 mL from liposome suspension and filtrate) was digested by sulfuric acid and then was oxidized to inorganic phosphate through incubating the samples in the presence of H_2_O_2_. The phosphomolybdic complex was formed upon interaction with ammonium molybdate. The complex was then reduced to a blue compound through interaction with 4-amino-3-hydroxy-1-naphthalenesulfonic acid. The absorbance of the blue compound was recorded at a wavelength of 815 nm. The following equation was used to calculate the incorporation rate (IR) of phospholipids:

(2) $${\mathrm{IR}}_{{\mathrm{PO}}_{4}^{3-}}\;(\%)=\frac{m_{{{\mathrm{PO}}_{4}^{3-}}_{T}}\;–m_{{{\mathrm{PO}}_{4}^{3-}}_{F}}\;}{m_{{{\mathrm{PO}}_{4}^{3-}}_{\mathrm{organic}\;\mathrm{phase}}}}\;\times \;100\;$$

where $m_{{{\mathrm{PO}}_{4}^{3-}}_{T}}\;$and$m_{{{\mathrm{PO}}_{4}^{3-}}_{F}}$ correspond to the total and free masses of phospholipids in the liposome suspension, respectively. $m_{{{\mathrm{PO}}_{4}^{3-}}_{\mathrm{organic}\;\mathrm{phase}}}$ is the mass of phospholipids initially added to the organic phase during the fabrication of liposomes.

##### Determination of Cholesterol Incorporation Rate

Chol dosage in the liposome formulations was performed using Chol CHOD-POD kit as previously described [26,40]. Briefly, 1 mL of the standard kit was added to 10 μL of each sample (Chol standards prepared in triton X-100, liposome suspension, and filtrate). The absorbance of the colored complex was measured at 505 nm. This equation was used to determine the IR of Chol:

(3) $${\mathrm{IR}}_{\mathrm{CHO}}\;(\%)=\frac{m_{{\mathrm{CHO}}_{\;T\;}}–{\;m}_{{\mathrm{CHO}}_{\;F}}}{m_{{\mathrm{CHO}}_{\;\mathrm{organic}\;\mathrm{phase}}}}\;\times \;100$$

where $m_{{\mathrm{CHO}}_{\;T\;}}$is the mass of Chol in the liposome suspension, $m_{{\mathrm{CHO}\;}_{F}}$ is the mass of Chol in the filtrate, $m_{{\mathrm{CHO}}_{\;\mathrm{organic}\;\mathrm{phase}}}$ and is the mass of Chol initially added to the organic phase during liposome production.

##### Quantification of α-Pinene in the Formulations

The total and free concentrations of α-pinene in the formulations were determined by the HPLC method described above. The encapsulation efficiency (EE) and loading rate (LR) values of α-pinene were calculated as follows:

(4) $$\mathrm{EE}\;(\%)=\frac{\left[\alpha -\mathrm{pinene}\right]{\;}_{\mathrm{total}\;}–\;\left[\alpha -\mathrm{pinene}\right]{\;}_{\mathrm{free}\;}}{\;{\left[\alpha -\mathrm{pinene}\right]}_{\;\mathrm{total}}}\;\times \;100\;$$

where $\left[\alpha -\mathrm{pinene}\right]{\;}_{\mathrm{total}\;}$and $\left[\alpha -\mathrm{pinene}\right]{\;}_{\mathrm{free}\;}$ correspond to the total and free α-pinene concentrations in liposomal suspension, respectively.

(5) $${\mathrm{LR}}_{\;}(\%)=\frac{m_{\mathrm{liposomal}\;\mathrm{suspension}}-{\;m}_{\mathrm{filtrate}}}{m_{\;\mathrm{initial}}}\;\times \;100$$

where ${m\;}_{\mathrm{liposomal}\;\mathrm{suspension}}$ stands for the mass of α-pinene in the liposomal suspension (total), and ${m\;}_{\mathrm{filtrate}}$ stands for the mass of α-pinene in the liposomal filtrate (free). For α-pinene-CLs, ${m\;}_{\mathrm{initial}}$ is the initial mass of α-pinene added to the organic phase during the fabrication of vesicles. For α-pinene-DCLs, ${m\;}_{\mathrm{initial}}$ is the initial mass of α-pinene used to prepare the HP-β-CD/α-pinene inclusion complex.

#### 3.6.3. In Vitro Release Study

Dialysis tubing was used to study the in vitro release of α-pinene from the solution of HP-β-CD/α-pinene inclusion complex and from the suspension of Lipoid S100-CLs. The dialysis membranes with a molecular weight cut-off (MWCO) of 0.5–1 kDa and 8–10 kDa were first soaked in water for 30 min at room temperature. The filtrate containing the HP-β-CD/α-pinene inclusion complexes was placed in the dialysis membrane (MWCO 0.5–1 kDa). For Lipoid S100 liposome formulations, the unretained α-pinene molecules were first removed by centrifugation and the α-pinene-loaded liposomes were resuspended in water and then introduced into the dialysis tube (MWCO 8–10 kDa). The dialysis tubes were put in 500-mL bottles containing distilled water, and the dialysates were stirred at 200 rpm. At predetermined time points, aliquots of 100 μL were removed from the dialysis tubes for the HPLC assay, as described previously. The percentage of α-pinene released from the formulations was calculated based on the following equation:

(6) $$\mathrm{Percentage}\;\mathrm{of}\;\mathrm{released}\;\alpha -\mathrm{pinene}\;(\%)=\frac{\left[\alpha -\mathrm{pinene}\right]{\;}_{t_{0}}-{\left[\alpha -\mathrm{pinene}\right]}_{t}\;}{\left[\alpha -\mathrm{pinene}\right]{\;}_{t_{0}}}\;\times \;100\;$$

where $\left[\alpha -\mathrm{pinene}\right]{\;}_{t_{0}}$ is the initial concentration of α-pinene determined in the dialysis tube and$\;{\left[\alpha -\mathrm{pinene}\right]}_{t}$ is the concentration of α-pinene determined at time t.

#### 3.6.4. DPPH Radical Scavenging Activity Measurements

The 2,2-diphenyl-1-picrylhydrazyl (DPPH) radical scavenging activities of α-pinene and α-pinene-loaded Lipoid S-100 liposomes were measured according to the method reported by Brand-Williams, Cuvelier, and Berset (1995) [63] with some modifications. The DPPH free radical scavenging activity measures the ability of the sample to donate hydrogen to the DPPH radical, resulting in bleaching of the stable DPPH free radical from the purple color of the DPPH cation to the yellow-colored diphenylpricryhydrazine. The greater the bleaching action observed, the higher the antioxidant activity. In brief, 1 mL of free α-pinene ethanolic solution (200 µg/mL) or α-pinene-loaded Lipoid S-100 liposomes (200 µg/mL) was mixed with 2 mL of ethanolic DPPH• solution (0.125 mM). The mixtures were kept in the dark for 1 h at room temperature and then centrifuged at 15,000 rpm for 30 min. The supernatant was collected and the decrease in free radical concentration was monitored by reading absorbance at 517 nm using a UV–vis spectrophotometer (U-2900, Hitachi High-Technologies, Shanghai, China). DPPH has a deep violet color in solution, and it becomes colorless or pale yellow as it is neutralized by antioxidants. The blank sample of free α-pinene was prepared by mixing 2 mL of ethanolic DPPH• solution with 1 mL of ethanol. The mixture containing 1 mL of blank liposomes and 2 mL of ethanolic DPPH• solution was used as a blank sample of α-pinene-loaded liposomes. The percentage of DPPH• scavenging activity was calculated using the following equation:

(7) $$\mathrm{Scavenging}\;\mathrm{activity}\;(\%)=(1-\frac{A_{s}}{A_{0}}\;)\;\times \;100\;$$

where $A_{s}$ and $A_{0}$ are the absorbance of sample and blank sample, respectively.

#### 3.6.5. Storage Stability

The stability of the various nanoparticles was investigated by assessing their mean particle size after 3 months of storage at 4 °C. Additionally, the percentage of remaining α-pinene in the whole suspensions was calculated after 3 months by the following equation:

(8) $$\mathrm{Percentage}\;\mathrm{of}\;\mathrm{remaining}\;\alpha -\mathrm{pinene}=\frac{{\left[\alpha -\mathrm{pinene}\right]}_{3\;\mathrm{months}}}{{\left[\alpha -\mathrm{pinene}\right]}_{t_{0}}}\;\times \;100$$

in which ${\left[\alpha -\mathrm{pinene}\right]}_{t_{0}}\text{and }{\left[\alpha -\mathrm{pinene}\right]}_{3\;\mathrm{months}}$ correspond to the total concentration of α-pinene in the liposome suspension determined immediately after preparation and after storage for 3 months at 4 °C, respectively.

### 3.7. Statistical Analysis

Statistical analysis was performed using the students’ *t*-test. All assays were carried out in triplicate. The results are expressed as the mean values ± standard deviation. The significance level was set at *p* ≤ 0.05.

## 4. Conclusions

In this paper, we studied the encapsulation of the essential oil component, α-pinene, into CLs and DCLs using hydrogenated (Phospholipon 90H) or non-hydrogenated (Lipoid S100) phospholipids and Chol. The size, the IR of phospholipids and Chol, the EE and LR of α-pinene, α-pinene release, and the storage stability of the different formulations were characterized. The DPPH radical scavenging activity of free α-pinene and α-pinene-loaded Lipoid S-100 liposomes was also evaluated. The incorporation of α-pinene was more efficient in Lipoid S100-CLs compared to the other formulations, and this is associated with a greater effect of α-pinene on lipid bilayer and particle size. Lipoid S100-liposomes presented a controlled release of α-pinene over time and maintained the DPPH• scavenging activity of α-pinene. In addition, the various CL and DCL batches were stable after three months of storage at 4 °C. Therefore, it would be valuable to study the antimicrobial and insecticidal effect of the α-pinene-loaded liposome for their application in agriculture and food products.

## Figures and Tables

**Table 1:** The complexation efficiency of α-pinene into HP-β-CD as a function of HP-β-CD:α-pinene molar ratio.

HP-β-CD:α-Pinene Molar Ratio	CE (%)
1:1	14.3 ± 1.3
2.5:1	40.6 ± 3.5
5:1	65.4 ± 6.2
7.5:1	80.6 ± 7.7
10:1	76.9 ± 3.8

**Table 2:** Characteristics of blank CLs, α-pinene-CLs, blank DCLs, and α-pinene-DCLs prepared with Phospholipon 90H or Lipoid S100. The values of size in italic are those obtained after 3 months of storage at 4 °C.

Size Distribution							Vesicle Characterization					
Sample	Population 1		Population 2		Population 3		IR of PL (%)	IR of Chol (%)		EE of α-Pinene (%)	LR of α-Pinene (%)	Final PL:Chol: α-Pinene Molar Ratio
	%	Mean Size (nm)	%	Mean Size (µm)	%	Mean Size (µm)						
Phospholipon 90H:Chol formulations												
Blank CLs	87.7 ± 6.7 *87.3 ± 6.5*	150 ± 0.0 *150 ± 0.0*	12.3 ± 6.7 *12.7 ± 6.5*	6.2 ± 0.9 *6.2 ± 0.5*	-	-	89.5 ± 6.3		75.9 ± 3.5	-	-	122:98:0
α-pinene-CLs	84.3 ± 7.2 *82.5 ± 11.6*	140 ± 13.4 *123 ± 25.4*	15.7 ± 7.2 *17.5 ± 11.6*	7.7 ± 0.0 *7.7 ± 0.0*	-	-	88.6 ± 6.0		76.3 ± 4.3	100.0 ± 0.0	0.2 ± 0.01	124:98:0.36
Blank DCLs	72.3 ± 7.3 *61.0 ± 5.8*	140 ± 10.9 *131 ± 0.0*	27.7 ± 7.3 *17.7 ± 1.3*	6.1 ± 0.8 *6.6 ± 0.2*	- *21.3 ± 7.1*	- *48.3 ± 4.8*	66.5 ±3.9		41.1 ± 1.3	-	-	90:59:0
α-pinene-DCLs	82.5 ± 1.9 *74.3 ± 2.2*	140 ± 10.9 *115 ± 17.8*	17.5 ± 1.9 *10.8 ± 1.3*	5.7 ± 0.4 *5.8 ± 0.6*	- *14.9 ± 2.0*	- *83.5 ± 3.5*	87.8 ± 3.8		63.2 ± 3.1	72.9 ± 6.8	0.2 ± 0.02	118:82:0.31
Lipoid S100:Chol formulations												
Blank CLs	17.0 ± 1.4 *14.2 ± 0.3*	166 ± 33.3 *172 ± 41.1*	83.0 ± 1.4 *85.8 ± 0.3*	6.7 ± 0.0 *8.4 ± 2.9*	-	-	95.9 ± 1.5		71.2 ± 5.4	-	-	119:92:0
α-pinene-CLs	-	-	33.4 ± 5.3 *12.3 ± 2.5*	9.7 ± 0.7 *10.6 ± 0.8*	66.6 ± 5.3 *87.6 ± 2.5*	83.5 ± 8.7 *84.8 ± 6.4*	48.6 ± 2.4		66.4 ± 4.9	100.0 ± 0.0	22.9 ± 2.2	61:93:42
Blank DCLs	-	-	-	-	100 ± 0.0 *100 ± 0.0*	26.1 ± 0.0 *34.2 ± 4.7*	83.1 ± 0.7		74.5 ± 1.7	-	-	103:100:0
α-pinene-DCLs	34.0 ± 0.0 *18.5 ± 2.2*	131 ± 8.3 *296 ± 9.7*	66.0 ± 0.0 *61.0 ± 2.0*	6.8 ± 2.9 *7.7 ± 1.9*	- *20.5 ± 1.4*	- *29.9 ± 1.8*	89.2 ± 1.4		71.2 ± 1.3	100.0 ± 0.0	0.6 ± 0.02	111:92:1

Chol: cholesterol; CLs: conventional liposomes; DCLs: drug-in-cyclodextrins-in-liposomes; EE: encapsulation efficiency; IR: incorporation rate; LR: loading rate; PL: phospholipids.

## Supplementary Materials

The following are available online, Figure S1: The particle size distribution plot of blank phospholipon 90H liposomes (A), α-pinene-loaded Phospholipon 90H liposomes (B), blank phospholipon 90H DCLs (C), α-pinene-loaded Phospholipon 90H DCLs (D), blank Lipoid S-100 liposomes (E), α-pinene-loaded Lipoid S-100 liposomes (F), blank Lipoid S-100 DCLs (G), α-pinene-loaded Lipoid S-100 DCLs (H).
