# Supplementary material for: Encapsulation of α-Pinene in Delivery Systems Based on Liposomes and Cyclodextrins
Source: Molecules. 2021 Nov 12;26(22):6840. doi: 10.3390/molecules26226840 (PMC8623189; doi:10.3390/molecules26226840)
Supplement: Supplementary file 1 [file molecules-26-06840-s001.zip › molecules-1453038-supplementary.pdf]

Supplementary materials

Encapsulation of α-pinene in delivery systems based on liposomes and cyclodextrins

Zahraa Hammoud[1,2], Maya Kayouka[1], Adriana Trifan[3], Elwira Sieniawska[4*], Jouda Mediouni Ben Jemâa[5], Abdelhamid Elaissari[2], Hélène Greige-Gerges[1*]

[1] Bioactive Molecules Research Laboratory, Doctoral School of Sciences and Technologies, Faculty of Sciences, Section II, Lebanese University, Lebanon; zahraahammoud93@gmail.com (ZH); maya.kayouka@gmail.com; (MK); helenegreige73@gmail.com (HGG)

[2] Univ Lyon, University Claude Bernard Lyon-1, CNRS, LAGEP-UMR 5007, F-69622 Lyon, France; elaissari@lagep.univ-lyon1.fr (AE)

[3] Department of Pharmacognosy, Faculty of Pharmacy, Grigore T. Popa University of Medicine and Pharmacy of Iasi, Iasi, Romania; adriana_trifan@yahoo.com

[4] Department of Natural Products Chemistry, Medical University of Lublin, 20-093, Lublin, Poland; esieniawska@pharmacognosy.org

[5] Laboratory of Biotechnology Applied to Agriculture, National Agricultural Research Institute of Tunisia (INRAT), University of Carthage, Tunisia; j_mediouni@hotmail.fr

* Correspondence： esieniawska@pharmacognosy.org (ES); helenegreige73@gmail.com (HGG)

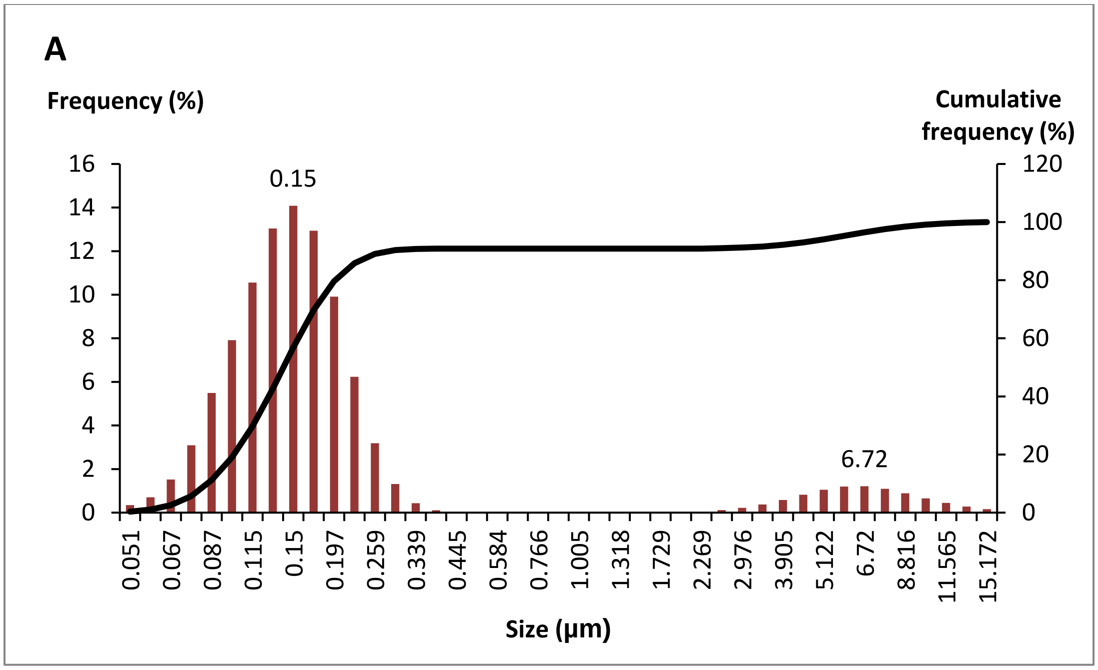
A
Frequency (%)
Cumulative frequency (%)
16
14
12
10
8
6
4
2
0
120
100
80
60
40
20
0
0.15
6.72
0.051
0.067
0.087
0.115
0.15
0.197
0.259
0.339
0.445
0.584
0.766
1.005
1.318
1.729
2.269
2.976
3.905
5.122
6.72
8.816
11.565
15.172
Size (μm)

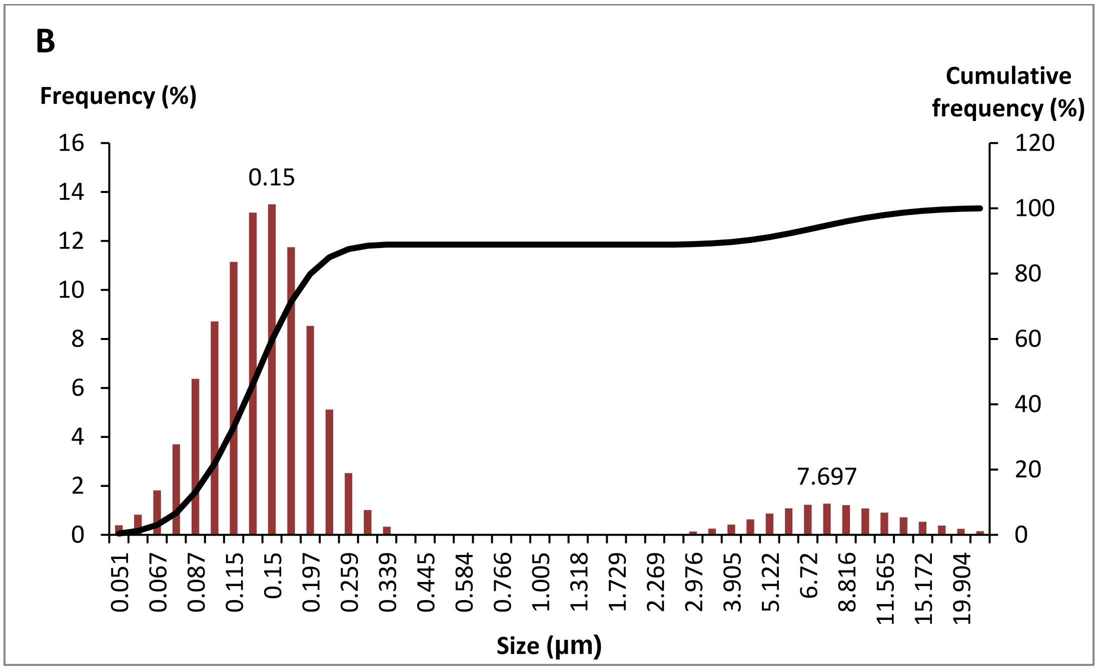
B
Frequency (%)
Cumulative frequency (%)
16
14
12
10
8
6
4
2
0
120
100
80
60
40
20
0
0.15
7.697
0.051
0.067
0.087
0.115
0.15
0.197
0.259
0.339
0.445
0.584
0.766
1.005
1.318
1.729
2.269
2.976
3.905
5.122
6.72
8.816
11.565
15.172
19.904
Size (μm)

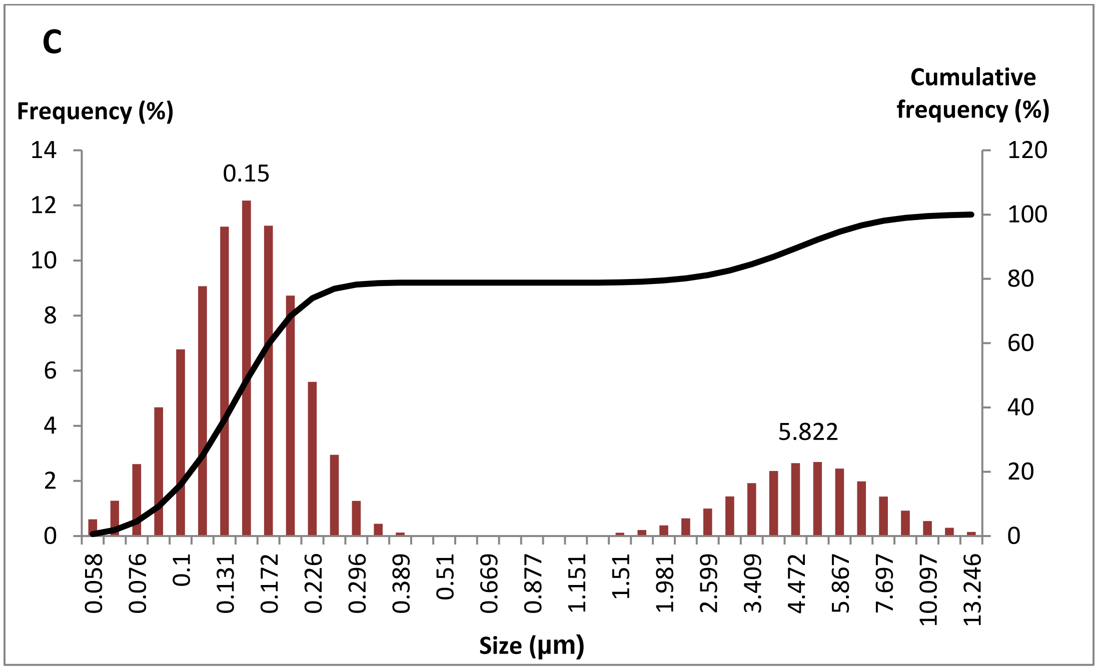
C
Frequency (%)
Cumulative frequency (%)
0.15
5.822
Size (μm)

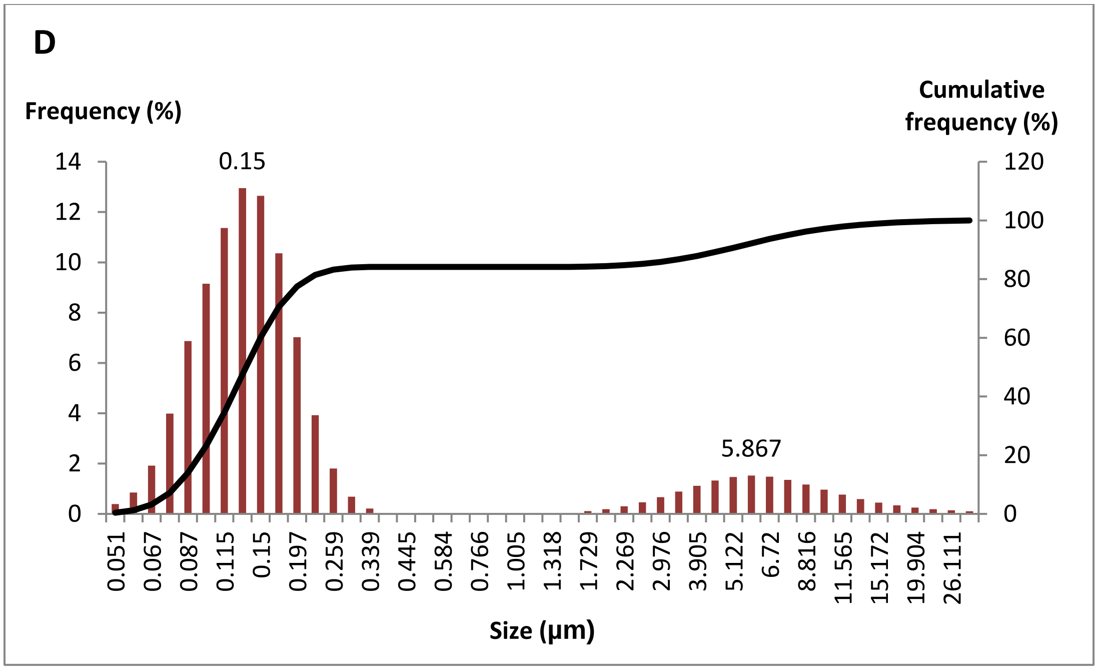
D
Frequency (%)
Cumulative frequency (%)
0.15
5.867
Size (μm)

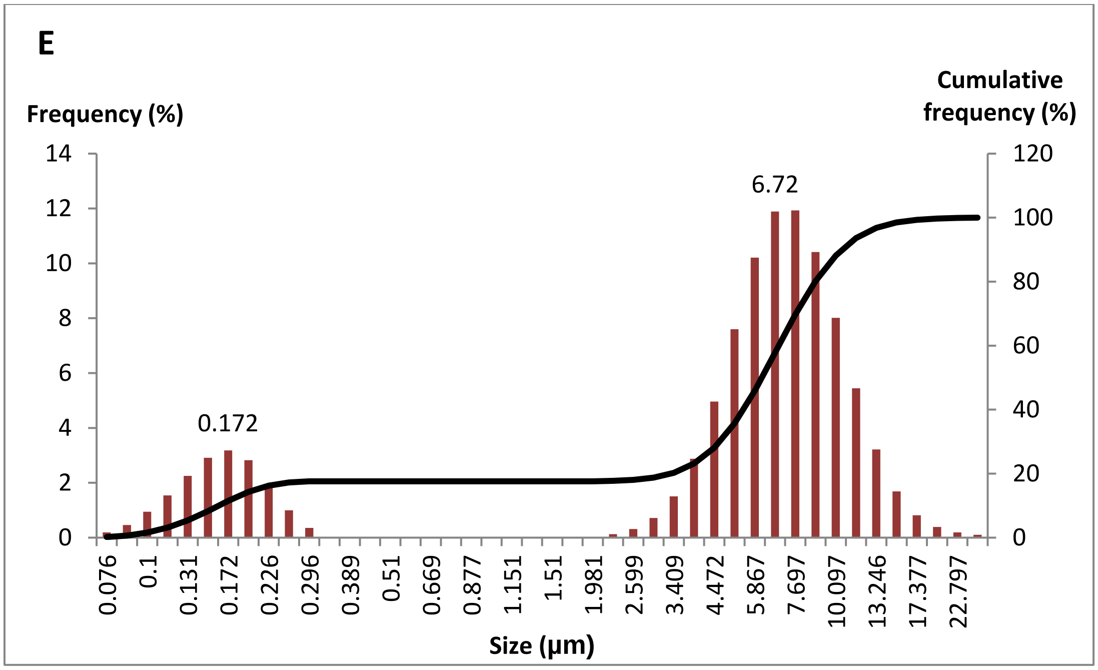
E
Frequency (%)
Cumulative frequency (%)
14
12
10
8
6
4
2
0
120
100
80
60
40
20
0
6.72
0.172
0.076
0.1
0.131
0.172
0.226
0.296
0.389
0.51
0.669
0.877
1.151
1.51
1.981
2.599
3.409
4.472
5.867
7.697
10.097
13.246
17.377
22.797
Size (μm)

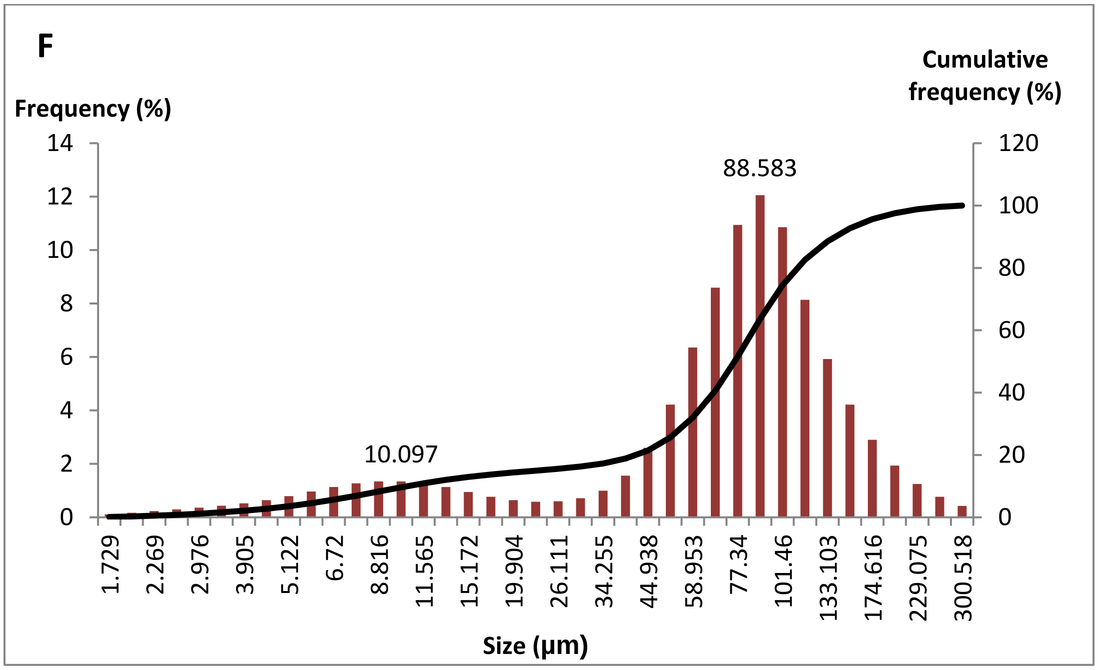
F
Frequency (%)
Cumulative frequency (%)
14
12
10
8
6
4
2
0
120
100
80
60
40
20
0
88.583
10.097
1.729
2.269
2.976
3.905
5.122
6.72
8.816
11.565
15.172
19.904
26.111
34.255
44.938
58.953
77.34
101.46
133.103
174.616
229.075
300.518
Size (μm)

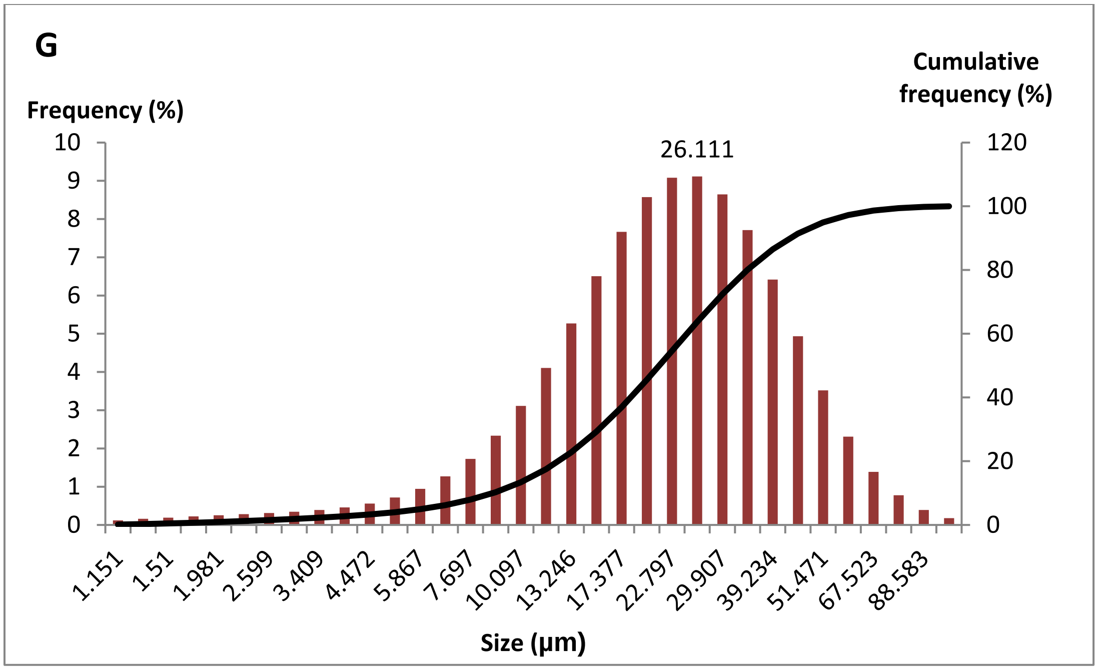

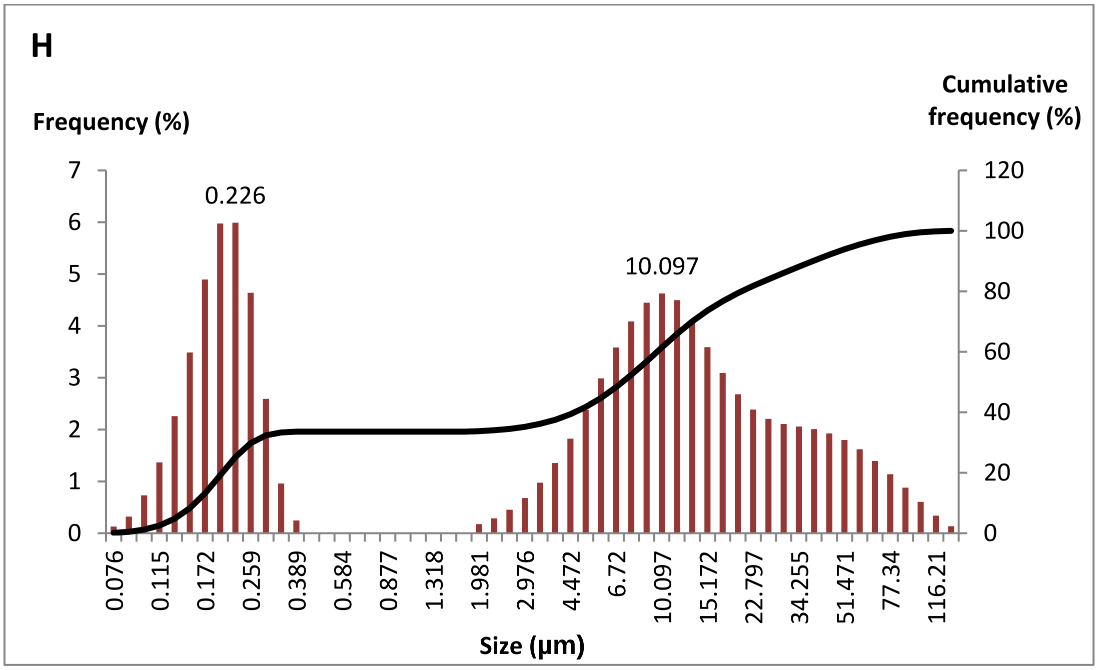

Figure S1: The particle size distribution plot of blank phospholipon 90H liposomes (A), α-pinene-loaded Phospholipon 90H liposomes (B), blank phospholipon 90H DCLs (C), α-pinene-loaded Phospholipon 90H DCLs (D), blank Lipoid S-100 liposomes (E), α-pinene-loaded Lipoid S-100 liposomes (F), blank Lipoid S-100 DCLs (G), α-pinene-loaded Lipoid S-100 DCLs (H).
